# ‘Know thyself’ – host factors influencing cancer response to immune checkpoint inhibitors

**DOI:** 10.1002/path.5907

**Published:** 2022-05-20

**Authors:** Ashray Gunjur, Andrea J Manrique‐Rincón, Oliver Klein, Andreas Behren, Trevor D Lawley, Sarah J Welsh, David J Adams

**Affiliations:** ^1^ Experimental Cancer Genetics, Wellcome Sanger Institute Hinxton UK; ^2^ Olivia Newton‐John Cancer Research Institute La Trobe University School of Cancer Medicine Heidelberg Australia; ^3^ Cambridge Institute of Therapeutic Immunology & Infectious Disease, Department of Medicine University of Cambridge Cambridge UK; ^4^ Department of Medical Oncology Austin Health Heidelberg Australia; ^5^ Department of Medicine University of Melbourne Parkville Australia; ^6^ Microbiotica Limited Cambridge UK; ^7^ Department of Surgery University of Cambridge Cambridge UK; ^8^ Cambridge University Hospitals NHS Foundation Trust Cambridge UK

**Keywords:** immune checkpoint inhibitors, immunotherapy, biomarkers, predictive, host, germline, immune system, microbiome, metabolome

## Abstract

Immune checkpoint inhibitors (ICIs) have revolutionised oncology and are now standard‐of‐care for the treatment of a wide variety of solid neoplasms. However, tumour responses remain unpredictable, experienced by only a minority of ICI recipients across malignancy types. Therefore, there is an urgent need for better predictive biomarkers to identify *a priori* the patients most likely to benefit from these therapies. Despite considerable efforts, only three such biomarkers are FDA‐approved for clinical use, and all rely on the availability of tumour tissue for immunohistochemical staining or genomic assays. There is emerging evidence that host factors – for example, genetic, metabolic, and immune factors, as well as the composition of one's gut microbiota – influence the response of a patient's cancer to ICIs. Tantalisingly, some of these factors are modifiable, paving the way for co‐therapies that may enhance the therapeutic index of these treatments. Herein, we review key host factors that are of potential biomarker value for response to ICI therapy, with a particular focus on the proposed mechanisms for these influences. © 2022 The Authors. *The Journal of Pathology* published by John Wiley & Sons Ltd on behalf of The Pathological Society of Great Britain and Ireland.

## Introduction

Though previously underappreciated, the relationship between cancer and host immunity is now fundamental to modern oncology practice. This has been catalysed by the discovery of ‘immune checkpoints’, immune self‐tolerance pathways that cancer may leverage to ‘placate’ the immune system and avoid immunological rejection [1]. Their breakthrough discoveries have led to the development of monoclonal antibody inhibitors capable of preventing this immune escape, sparking an ongoing ‘immuno‐oncology revolution’. In 2011, the US Food and Drug Administration (FDA) approved the first immune checkpoint inhibitor (ICI) targeting cytotoxic T‐lymphocyte‐associated protein 4 (CTLA‐4 or CD152), after it became the first drug ever shown to improve survival for patients with advanced melanoma [2]. Since then, ICIs targeting programmed cell death protein 1 (PD1 or CD279) and its ligand (PDL1 or CD274) have been approved for a varied and ever‐growing list of solid‐organ malignancies including melanoma, non‐small cell lung carcinoma (NSCLC), renal cell carcinoma (RCC), and urothelial carcinoma, amongst many others [3].

However, important caveats have tempered the success of ICIs. Firstly, in the blockade of these immune homeostatic pathways, a subset of patients will develop auto‐immune or auto‐inflammatory disease, collectively labelled ‘immune‐related adverse events’ (irAEs). The patterns and proportions vary by ICI class, with reports of clinically significant irAEs for metastatic melanoma patients of approximately 15%, 20%, and 55% for anti‐PD1, anti‐CTLA4, and combination ICI therapy respectively [4, 5]. Secondly, though tumour responses to ICIs are often durable and clinically meaningful, they too are capricious and only occur in approximately 10–50% of patients with differing tumour histologies [3]. As such, extensive work has been invested in defining pre‐treatment biomarkers: reliably‐assessed biological signs that predict *a priori* who will clinically benefit. Thus far, only three such biomarkers have been approved by the FDA for clinical use: namely, tumour tissue PDL1 protein, tumour mutational burden (TMB), and mismatch repair (MMR) deficiency [6]. Unfortunately, both PDL1 and TMB are limited by issues of disharmony between assays, variable relevance across cancer types, and poor specificity (with responses still observed when the assay is deemed ‘negative’ and vice versa) [7, 8]. Though MMR deficiency powerfully enriches for ICI response, it occurs in less than 5% of advanced cancers, limiting its applicability [9]. Further work has involved exploring characteristics of the tumour immune microenvironment, including tumour‐infiltrating lymphocytes (TILs), innate immune cell characteristics, and immune gene expression scores. However, none have been approved for clinical use [10].

Notably, the bulk of ICI biomarker discovery efforts are ‘tumour‐centric’, and are thus fundamentally reliant on tumour tissue. This may necessitate a further invasive procedure for patients where no contemporary archival tumour tissue is available, introducing their associated risk and the possibility of sampling error due to intralesional heterogeneity [11]. Additionally, tumour‐centric assays are less likely to be generalisable across cancer types – important when we consider the ever‐expanding indications for ICI therapy. For example, we observed that TMB failed to associate with the responsiveness of a mixed cohort of advanced biliary tree, neuroendocrine, and rare gynaecological cancers treated with combination ICIs [12]. Just as ICI efficacy relies on the interface of the tumour and host immunity, we envision that optimal *a priori* prediction of ICI responsiveness will require consideration of both tumour and host features, for example, using ‘immunogram’‐like approaches [13].

**Figure 1:**
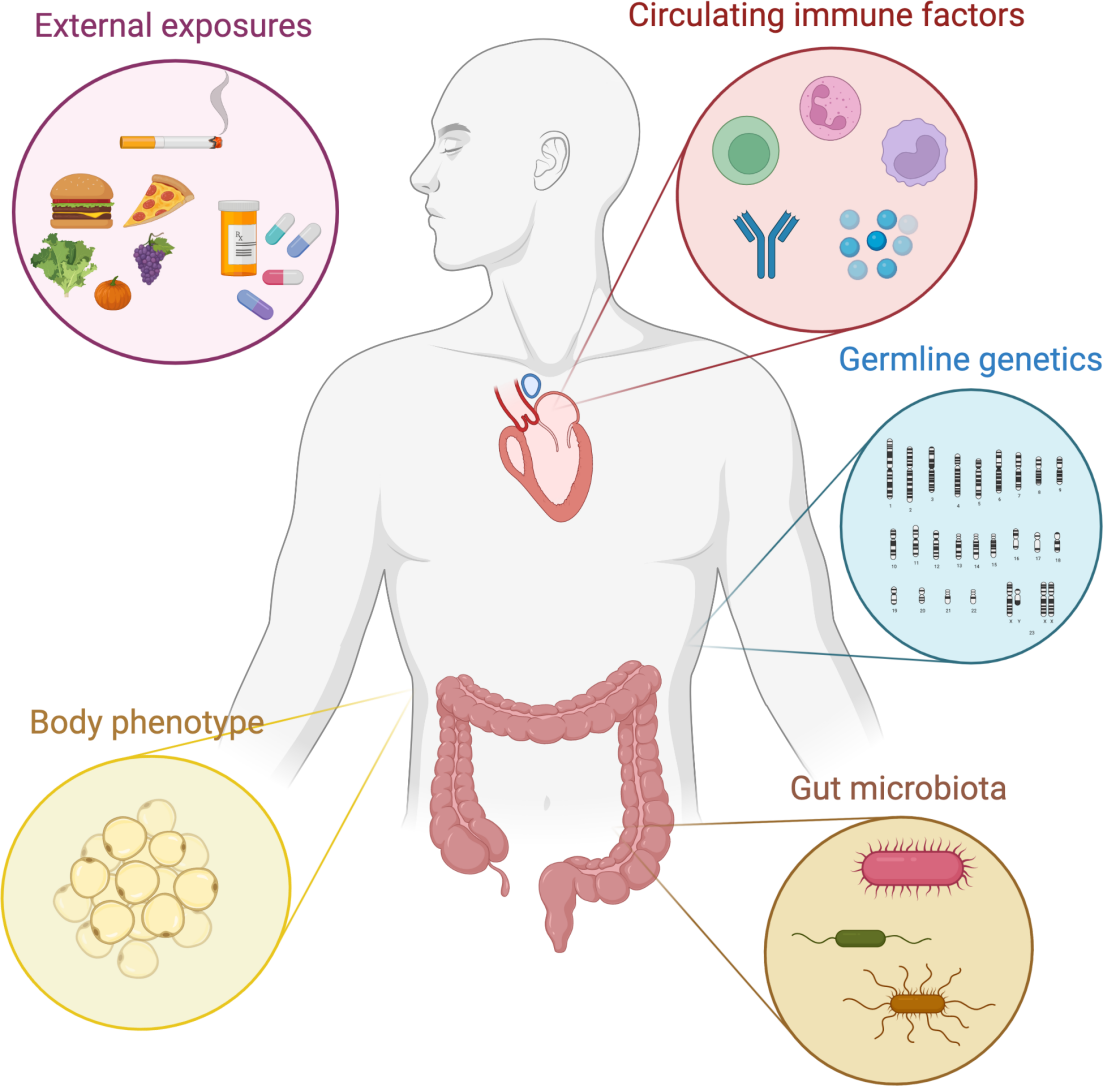
Overview of the host factor domains discussed.

Therefore, in this review we highlight the evidence supporting a myriad of host‐based factors as potential features in future ICI biomarker discovery efforts (Figure 1). We begin by reviewing circulating immune factors that may have pre‐treatment prognostic or predictive value. We then describe germline genetic traits as well as general phenotypic host factors (such as body habitus and gender) that have been implicated in ICI response. Additionally, we briefly summarise evidence supporting the relevance of key exogenous factors (concurrent medications and diet) in modulating host immunity, and thus ICI efficacy. Finally, we end by reviewing the growing literature connecting the composition and diversity of our gut microbiota and ICI efficacy, complemented by a thorough discussion of the potential mechanisms for this relationship that have thus far been elucidated.

## The circulating immune compartment

A prerequisite of anti‐cancer immunity (and ICI efficacy) is the recruitment of immune cells to the tumour from primary and secondary lymphoid organs, facilitated by the release of signalling molecules (cytokines) and involving diverse immune cell populations [14]. Indeed, peripheral blood contains a complex milieu of diverse white blood cells (WBCs) and soluble factors whose quantities may indirectly reveal a cancer's ‘immune phenotype’ [15]. As such, there has been considerable interest in whether their baseline measurement might insinuate a cancer's susceptibility to ICI therapy.

Peripheral WBCs may be classified morphologically, with neutrophils and lymphocytes usually the most abundant subtypes. A high baseline blood neutrophil‐to‐lymphocyte ratio (NLR) has long been noted to confer a negative prognosis, irrespective of cancer or therapy [16]. This negative relationship holds true for ICI‐therapy recipients; for example, high pre‐treatment NLR was associated with worse overall survival (OS), progression‐free survival (PFS), and objective response rate (ORR) in a large, pan‐cancer cohort [17]. Biologically, peripheral neutrophilia may correlate with tumour microenvironment (TME) neutrophil infiltration [18], where they might act to suppress anti‐cancer T‐cell trafficking. Supporting this, Kargl *et al* found an inverse relationship between infiltrating neutrophils and CD8‐expressing T cells (CD8^+^ T cells) in NSCLC tumour samples [19], with their subsequent analysis linking this intratumoural NLR to poor ICI efficacy [20]. Using a murine lung cancer model, they demonstrated that neutrophil antagonism restored tumour CD8^+^ T cell infiltration as well as anti‐PD1 efficacy [20].

Conversely, pre‐treatment eosinophilia appears to be associated with better outcomes in retrospective analyses of anti‐PD1‐ [21] and anti‐CTLA4‐treated [22, 23] melanoma and anti‐PD1‐treated NSCLC patients [24], and lower neutrophil‐to‐eosinophil ratio correlated with outcomes for combination anti‐PD1/anti‐CTLA4‐treated metastatic RCC patients [25]. Preclinically, Carretero *et al* demonstrated that eosinophils play a key role in attracting CD8^+^ T cells through the release of chemokines, which may mediate this increased ICI susceptibility [26].

Flow and mass cytometry revealed relevant associations between certain WBC subtypes and ICI efficacy, such as high baseline T regulatory cells (Tregs) (FoxP3^+^CD4^+^ T cells) associating with ipilimumab (anti‐CTLA‐4) efficacy [22] and classical (CD14^+^CD16^−^) monocytes associating with anti‐PD1 efficacy in melanoma [27]. The high dimensionality of mass cytometry data has also led to more complicated WBC response ‘signatures’ being defined [28], with efforts underway to harmonise biomarker panels for future work [29]. Next‐generation sequencing (NGS) of sorted peripheral WBC subtypes has increased this dimensionality of data even further, with intriguing signals regarding its utility in predicting ICI efficacy. For example, the baseline and dynamic significance of peripheral blood T‐cell receptor (TCR) repertoire has garnered much interest; however, bulk NGS approaches have reached differing conclusions about their association with ICI susceptibility [30, 31, 32, 33]. By cell sorting before NGS, Gros *et al* observed in melanoma that the TCR repertoire of specifically the peripheral PD1^+^CD8^+^ T cell subset matched that found on TILs, suggesting these are tumour‐reactive T‐cell populations circulating in the peripheral WBC compartment [34]. Building on this, Han *et al* found an association between the TCR diversity of these cells and better disease control and PFS for NSCLC patients receiving anti‐PD(L)1 therapy, suggesting this assay's predictive biomarker potential [35].

Cytokines are essential mediators for intercellular communication and can confer pro‐ or anti‐tumourigenic climates, and, as such, have also been studied in relation to malignancy and ICI efficacy [36]. For example, interleukin (IL) 8 (encoded by *CXCL8*) is a potent pro‐inflammatory neutrophil and myeloid‐derived suppressor cell (MDSC) chemoattractant with a short circulating half‐life, known to reflect systemic tumour volume [37, 38]. In a large *post hoc* analysis of three trials testing atezolizumab (anti‐PDL1) for metastatic RCC or urothelial cancer, Yuen *et al* consistently found a negative correlation between high plasma IL‐8 and efficacy (OS or ORR). Through a single‐cell RNA sequencing analysis of a sub‐group of peripheral blood mononuclear cells (PBMCs) and tumour samples, *CXCL8* mRNA was associated most strongly with the myeloid compartment of both PBMCs and tumour samples, connecting it with myeloid‐mediated immune suppression [39].

Finally, associations are also emerging between baseline circulating auto‐antibodies and ICI efficacy, including those that are associated with auto‐immunity (such as rheumatoid factor) [40]. Further efforts have used wider auto‐antibody profiles, particularly incorporating tumour‐associated antibodies (TAAs) [41, 42, 43]. Two studies have associated baseline anti‐NY‐ESO‐1 with ICI efficacy for NSCLC patients, suggesting that this may be a relevant TAA. However, though intriguing, more work is needed to validate these associations, the mechanism underpinning them, and their generalisability across cancer types.

## Germline genetic features

The human leukocyte antigen class I (HLA‐I) complex is responsible for antigen presentation to CD8^+^ T cells, with its encoding genes (*HLA‐A*, ‐*B*, and ‐*C*) amongst the most highly polymorphic in the human genome. There is considerable variability in the peptide‐binding characteristics between *HLA* gene alleles, and it is therefore plausible that some may present cancer neoantigens more (or less) effectively than others. Recently, Naranbhai *et al* demonstrated that in a pan‐cancer cohort, harbouring one or two HLA‐A*03 alleles was associated with a poorer OS after ICI therapy [44]. They went on to externally validate this in multiple other pan‐cancer cohorts and importantly established its ‘predictiveness’ (i.e. ICI specificity) by finding no difference in OS for non‐ICI‐treated patients. However, these findings were not replicated in another recent pan‐cancer analysis of pembrolizumab (anti‐PD1)‐treated patients; thus, its clinical use remains investigational [45].

Other work has focused on more global HLA‐I attributes. Akin to reports in HIV [46], Chowell *et al* demonstrated an HLA‐I ‘heterozygote advantage’, whereby homozygosity for at least one HLA‐I gene was associated with worse OS in an advanced NSCLC and melanoma cohort [47]. Conversely, they found better OS in melanoma anti‐CTLA4 recipients harbouring an *HLA‐B44* supertype allele. Similarly, further work by the same group evaluated the relevance of HLA‐I evolutionary divergence (HED), a measure of the difference of the peptide binding sites of each HLA‐I allele [48]. They found that greater mean HED was associated with better OS and ORR in melanoma and NSCLC patients, consistent with the ‘divergent allele advantage’ theory (whereby more diverse HLA‐I allele pairs would plausibly present more diverse cancer neoantigens). However, the relationship of HLA‐I zygosity and/or mean HED and better ICI efficacy has not been consistently found in more recent analyses for non‐melanoma cohorts [45, 49, 50, 51]. Importantly, the HLA‐B44 supertype seemed to impart an opposite, negative effect in NSCLC, potentially due to NSCLC's distinct neoantigen landscape (leading to worse presentation on *HLA‐B44*) [52]. Therefore, caution must be applied when extrapolating HLA‐I associations between cancer types.

In a related vein, Manczinger *et al* developed a score of ‘HLA‐I promiscuity’ based on estimating the diversity of peptides binding to each individual's HLA‐I alleles. They found that HLA‐I promiscuity was associated with worse OS and ORR after ICI therapy, mediated by increased expression of immune tolerance genes intratumourally. This aligns with the theory that greater HLA‐I promiscuity limits the ability to distinguish self epitopes versus tumour neoepitopes [53]. Once again, HLA‐I promiscuity is partly informed by the variety of binding tumour neoepitopes, marrying with the concept of tumour ‘immune fitness’ and likely varying by tumour histology types [54].

Another immune receptor implicated in anti‐CTLA4 response is the Fc‐γ receptor (FcγR). Previous work implicates antibody‐dependent cell cytotoxicity of CTLA‐4‐positive Tregs by FcγR‐expressing immune cells as part of the mechanism of action of ipilimumab (an IgG1 anti‐CTLA4 construct) [55]. Concordantly, a V158F single nucleotide polymorphism (SNP) (rs396991) of *FCGR3A* (encoding FcγRIIIA) was associated with improved ORR and OS in ipilimumab‐treated melanoma cohorts, specifically for those tumours with higher insertion–deletion (indel) burden [56].

There are emerging signs that germline alleles associated with the development of autoimmune syndromes or cancer may also predict ICI efficacy. In a genome‐wide association study, Chat *et al* associated the rs17388568 SNP (related to colitis and type 1 diabetes) with melanoma anti‐PD1 response [57]. Similarly, several groups have associated ICI susceptibility with polygenic risk scores for rheumatoid arthritis [58], autoimmune thyroid, and dermatological conditions, respectively [59, 60]. Finally, germline polymorphisms of the genes encoding relevant immune checkpoints (*CTLA4*, *PDCD1*, and *PDL1*) have been implicated in cancer risk [61], with signals from small retrospective studies starting to emerge that particular alleles may also associate with ICI benefit [62, 63, 64, 65]. Though intriguing and biologically plausible, these associations need large‐scale clinical and experimental validation prior to further development as biomarkers for ICI efficacy.

## Phenotypic and external features

In 2018, a pooled analysis found an unexpected association between obesity (measured by body mass index; BMI) and better OS for anti‐CTLA4‐ or anti‐PD1/PDL1‐treated patients with advanced melanoma [66]. Since then, several other *post hoc* analyses have been published, with most (though not all) supporting an ‘obesity paradox’ for ICI efficacy [67]. Preclinical efforts demonstrated that for diet‐induced obese mice, engrafted B16 melanoma cells demonstrated a more aggressive phenotype with more glucose uptake and ulceration/necrosis than their lean counterparts [68]. Interestingly, this phenotype appeared to be mediated by leptin promoting PD1 expression on intratumoural CD8^+^ T cells. Consequently, the relative benefit of anti‐PD1 therapy was far greater in obese (versus lean) mice. In contrast, Murphy *et al* found no anti‐CTLA4 efficacy in Renca (RCC) engrafted leptin‐induced obese mice, potentially reflecting the differing targets of these ICI classes, or biological differences in the tumour models used [69].

A similar ‘cholesterol paradox’ is also emerging. For example, in a retrospective review of NSCLC patients receiving anti‐PD1 therapy, high total cholesterol correlated with both PFS and OS after adjustment for other covariates (such as gender, BMI, and smoking status) [70]. Intriguingly, this relationship was not seen in a chemotherapy‐treated cohort, suggesting potential ICI specificity. Like obesity, it was found that tumour cholesterol content also influenced a PD1^+^CD8^+^ T‐cell phenotype in murine melanoma TILs dose‐dependently, thus once again potentially providing more substrate for anti‐PD1 efficacy [71].

Gender may also be relevant to ICI efficacy. A *post hoc* meta‐analysis of 20 published randomised controlled trials of anti‐CTLA4 or anti‐PD1 therapies found that males appeared to derive significantly more relative benefit from ICIs, particularly anti‐PD1 therapies [72]. Like cholesterol and obesity, this may relate to rates of intratumoural CD8^+^ T‐cell exhaustion, with an analysis of pre‐treatment melanoma samples finding those from men exhibiting higher CD8^+^ TIL fractions strongly positive for CTLA4 and PD1. However, a more recent meta‐analysis (which included trials of anti‐PDL1 therapies, and more recent trials of combination chemo‐immunotherapy) did not find any significant interaction of gender on OS [73]. As such, the relationship between gender and ICI efficacy remains controversial.

Coined our ‘exposome’ [74], several exogenous and endogenous environmental factors may also interact with host immunity to modulate ICI efficacy. For example, concurrent medications are likely relevant, with exogenous corticosteroids being perhaps the most studied. *In vivo*, dexamethasone (a corticosteroid) was found to partially abrogate anti‐PD1 tumour growth inhibition by impairing peripheral CD8^+^ T‐cell responses [75], and clinically, a meta‐analysis of 16 trials found a negative association between their use and OS/PFS. Importantly, on sub‐group analysis by indication, this negative effect was only seen when they were used for supportive care, and not for those treating irAEs [76]. Conversely, similar *post hoc* clinical analyses have implicated a potential benefit to ICI efficacy from concurrent pan‐β‐blocker [77], statin [78, 79], and antihistamine use [80]. Though retrospective associations may be confounded by indication bias (for example, the cutaneous toxicity that prompts antihistamine use may itself be positively associated with ICI efficacy [81]), *in vivo* work has demonstrated that histamine may promote a pro‐tumour M2 macrophage phenotype intratumourally, biologically supporting the potential benefit of concurrent antihistamine therapy [80].

Finally, other medications (e.g. antibiotics [82], proton‐pump inhibitors [83]) or dietary choices (e.g. high‐fibre intake [84]) may impact ICI efficacy (negatively or positively, respectively) via influencing our gut microbiome – the composition and diversity of resident microbiota living in our gastrointestinal tract. As elaborated next, there is an emerging understanding of the relevance of the gut microbiome to systemic ICI efficacy, the mechanisms underpinning this, and its potential utility in selecting patients for these therapies.

## Gut microbial and related features

The human gut microbiota harbours ~10^14^ microbes and is dominated by bacteria from the Bacteroidetes, Firmicutes, Actinobacteria, and Proteobacteria phyla [85]. An adult harbours hundreds of species and hundreds of strains of anaerobic bacteria that provide beneficial properties impacting immunological development and regulation [86, 87]. Gut microbiome profiling with 16S rRNA sequencing (lower‐resolution taxonomic profiles, usually to genus level) and shotgun metagenomic sequencing (high‐resolution taxonomic profiling up to subspecies level and functional profiling) have linked its composition and functions to a growing list of cancers, and to cancer therapy efficacy.

Much of our early understanding linking gut microbiota and cancer therapies is based on studies in murine models. In 2013, Viaud *et al* demonstrated that the anti‐cancer immune effects of cyclophosphamide were reliant on the gut microbiota, with gut integrity disruption allowing microbial translocation into secondary lymphoid tissue and triggering systemic cell‐mediated immunity [88]. In 2015, *in vivo* work demonstrated how responses to anti‐CTLA4 and anti‐PD1 were predicated on the presence of enteric *Bacteroides* and *Bifidobacterium* genera, respectively [89, 90]. In 2018, concurrent publications linked the composition [91, 92, 93] and diversity [91] of baseline gut microbiota with anti‐PD1 response in diverse clinical cancer cohorts. Compellingly, each group would demonstrate the recapitulation of response or non‐response *in vivo* by faecal microbiota transplant (FMT) of human patient stool to mice engrafted with murine melanoma (B16.SIY and BP), sarcoma (MCA‐205), and renal cancer (Renca), providing evidence of causality and a link between human and murine systems. Finally, in 2021, the results of two phase I trials demonstrated this recapitulation human‐to‐human, with responders' stool reinvigorating anti‐PD1 response in a subset of melanoma patients with resistant or refractory tumours [94, 95].

**Figure 2:**
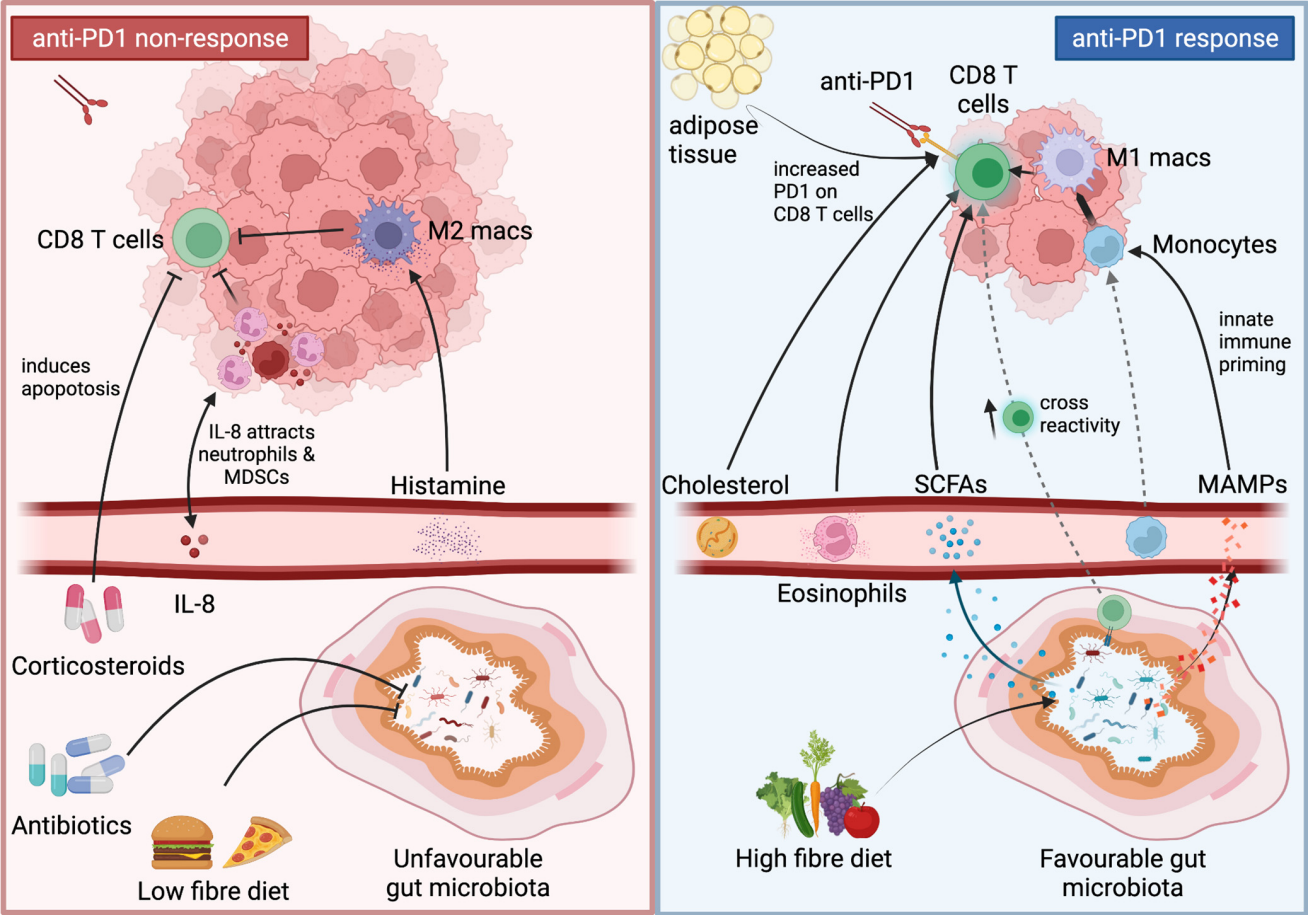
Potential mechanisms of host factor influence on anti‐PD1 non‐response/response. Left panel – anti‐PD1 non‐response. Corticosteroids: induce T‐cell apoptosis [75]. Antibiotics and a low‐fibre diet: promote an unfavourable gut microbiota milieu, reducing its positive impact on anti‐PD1 efficacy (pictured in the right panel) [84, 92]. Interleukin (IL) 8: produced by (and contributing to) tumour microenvironment (TME) infiltration of neutrophil and myeloid‐derived suppressor cells [39]. Histamine: promotes differentiation into M2 (pro‐tumour) macrophages (macs) within the TME [80]. Right panel – anti‐PD1 response. Cholesterol and adipose tissue: increase PD1 expression on CD8^+^ T cells [67, 71]. Eosinophils: attract CD8^+^ T cells to the TME [26]. A high‐fibre diet: promotes a favourable gut microbiota milieu, which: increases production of short‐chain fatty acids (SCFAs), which enhance CD8^+^ T‐cell anti‐tumour cytotoxicity [112]; allows cross‐priming of CD8^+^ T cells (‘molecular mimicry’) [113]; and stimulates innate immune receptors (e.g. NOD2, STING) that promote an anti‐tumour myeloid cell response [114, 115].

However, we observe relatively little consensus when we examine the annotated species correlated ‘positively’ and ‘negatively’ by studies profiling baseline stool in ICI recipients, both in meta‐analyses of the pivotal 2018 data using uniform bioinformatic pipelines [96, 97, 98] and in subsequent patient cohort publications (Table 1, with contradictory species‐efficacy associations underlined). These discrepancies could be due to differences in clinical (e.g. patient geography, cancer histology, ICIs used) or methodological approaches (e.g. stool collection and storage, DNA extraction and sequencing, clinical endpoints used). They could also be explained by the immunomodulatory properties of phylogenetically‐distant gut microbial species converging through common functions, such as common metabolite production. For example, Mager *et al* found that both *Bifidobacterium pseudolongum* and *Akkermansia muciniphila* might synergise with anti‐CTLA4 efficacy through their common production of inosine, which might help to shift tumour‐associated CD4^+^ T cells to a Th1 (anti‐tumour) phenotype by agonism of adenosine receptors [110]. Another explanation for the lack of a common microbiome signal between the various studies is that the clinical response may be due to complex combinations of evolutionarily distinct anaerobic bacteria. For instance, Tanoue *et al* recently found a combination of 11 specific strains, themselves a tiny contributor to average gut microbiome complexity, that could robustly induce cytotoxic T‐cell responses and enhance ICI efficacy [111]. As such, herein we focus on the potential mechanisms that may explain the powerful influence of gut microbiota on ICI efficacy (Figure 2).

**Table 1:** Clinical studies associating pre‐treatment gut bacteria species‐level taxonomic abundance (by shotgun metagenomic profiling) with ICI efficacy.

Reference	PY	Country	Histology	*n*	ICI	Positively associated species	Negatively associated species	Clinical endpoint
[99]	2017	USA	Melanoma	39	CICB (24), anti‐CTLA4 (1), anti‐PD1 (14)	*Bacteroides caccae*, *Streptococcus parasanguinis*	*Bacteroides eggerthii* , *Atopobium parvulum*	ORR
[91]	2018	USA	Melanoma	25	Anti‐PD1		*Anaerotruncus colihominis*, *Klebsiella variicola*, *Escherichia coli* , *Bacteroides thetaiotaomicron*	PFS6
[93]	2018	USA	Melanoma	39	Anti‐PD1	*Enterococcus faecium*, *Collinsella aerofaciens*, *Bifidobacterium adolescentis*, *Klebsiella pneumoniae*, *Veillonella parvula*, *Parabacteroides merdae*, *Bifidobacterium longum*	*Ruminococcus obeum*, *Roseburia intestinalis*	ORR
[92]	2018	France	NSCLC, RCC	100^†^	Anti‐PD1	*Akkermansia muciniphila* , *Enterococcus hirae*		PFS6
[100]	2019	China	HCC	8	Anti‐PD1	*Bifidobacterium dentium*, *A. muciniphila*	*Bacteroides nordii*	CR + PR + SD6
[101]	2019	USA	Melanoma	27	CICB (12), anti‐PD1 (14), anti‐CTLA4 (1)	*Faecalibacterium prausnitzii*	*Bacteroides ovatus*, *Blautia producta*, *Ruminococcus gnavus*	PFS
[102]	2020	The Netherlands	Melanoma	25	Anti‐PD1 (23), CICB (2)	*R. gnavus*, *E. coli* , *Eubacterium biforme*, *Bacteroides eggerthii*	*Bifidobacterium longum* , *A. muciniphila* , *Prevotella copri*, *S. parasanguinis*	CR + PR + SD3
[103]	2020	France	RCC	69^†^	Anti‐PD1	*A. muciniphila* , *Bacteroides salyersiae*	*Hungetella hathewayi*, *Clostridium clostridioforme*	CR + PR + SD6
[104]	2020	USA	RCC	31	CICB (7), anti‐PD1 (24)	*Bifidobacterium adolescentis*, *Barnesiella intestinihominis*, *Odoribacter splanchnicus*, *Bacteroides eggerthii*	*Bacteroides ovatus*	CR + PR + SD4
[105]	2020	China	Mixed GI cancers	40	CICB (14), anti‐PD1 (14), anti‐PDL1 (12)	*Eubacterium rectale*		CBR
[106]	2021	USA	Melanoma	38	CICB	*Bacteroides stercoris*, *Parabacteroides distasonis*		ORR
[107]	2021	China	HCC, BTC	65	Anti‐PD1	*F. prausnitzii*, *Prevotellamassilla timonensis*, *Gemmiger formicillis*, *Niabella drillacis*, *Lactobacillus rogosae*, *Eubacterium elligens*	*Parabacteroides distasonis* , *Citrobacter freundii*, *Haemophilus parainfluenzae*, *Dialister succinatiphillus*	CR + PR + SD6
[108]	2021	South Korea	HCC	8	Anti‐PD1	*Eubacterium coprostanoligenes*	*R. gnavus*	CR + PR + SD6
[84]	2021	USA	Melanoma	111	Anti‐PD1	*F. prausnitzii*		CR + PR + SD6
[109]	2022	France, Canada	NSCLC	338	Anti‐PD1	*A. muciniphila*		ORR
[98]	2022	UK	Melanoma	53	Anti‐PD1	*Bacteroides* *eggerthi*		ORR

BTC, biliary tree carcinoma; CICB, combined immune checkpoint blockade [anti‐CTLA4 + anti‐PD(L)1]; CR, complete response; GI, gastrointestinal; HCC, hepatocellular carcinoma; ICI, immune checkpoint inhibitor; NSCLC, non‐small cell lung carcinoma; ORR, objective response rate; PFS, progression‐free survival; PR, partial response; PY, publication year; RCC, renal cell carcinoma; SD, stable disease (addended number indicates a minimum duration in months).

Species underlined indicate taxa where different studies have found opposing associations with ICI efficacy.

* Where a cohort of patients treated with different ICIs were combinatorially analysed, the subset treated with each class is indicated in parentheses.

^†^ Overlap of 40 patients reported by these two papers.

As previously mentioned, gut bacteria often provide humans with beneficial functions through their metabolic products absorbed into our circulation, with the gut microbiome sometimes referred to as ‘the neglected endocrine organ’ [116]. The connection between the milieu of gut microbiota and circulating metabolites is consistently seen, with plasma levels of the microbial–host co‐metabolite hippurate appearing to be a particularly reliable marker of overall gut microbiome diversity [117, 118]. Interestingly, pre‐treatment blood hippurate levels correlate with anti‐PD1 efficacy, supporting a connection between a diverse gut microbiome and ICI efficacy [119]. Gut microbiota are capable of metabolising tryptophan, with levels of its metabolite kynurenine [120] and associated enzymes 3‐hydroxyanthranilic acid [121] and indoleamine‐pyrrole 2,3‐dioxygenase (IDO) [122] all inversely associated with ICI efficacy.

Short‐chain fatty acids are produced during bacterial fermentation of dietary fibre, and appear to be particularly relevant through their diverse immunomodulatory properties. Butyrate in particular has been shown, on the one hand, to enhance anti‐tumour CD8^+^ T‐cell function through increasing IL‐12 receptor [112] and memory T‐cell survival [123] *in vivo*. Concordantly, faecal butyrate was associated with better anti‐PD1 responses in a cohort of mixed histology patients [124]. On the other hand, butyrate has been shown to promote regulatory CD4^+^ T cells and impair dendritic cell maturation, thus negatively associating with anti‐CTLA4 efficacy [125].

Microbe‐associated molecular patterns (MAMPs) are molecules that are widely essential to (and thus conserved across) commensal microbiota. They are recognised by the innate immune system via a variety of pattern‐recognition receptors, with this interaction also likely affecting systemic anti‐cancer immunity. For example, Griffin *et al* isolated the anti‐PD1 synergistic effect of *Enterococcus faecium* to the *SagA* gene, responsible for peptidoglycan breakdown into muropeptides. Exogenous muropeptides recapitulated this effect only in the presence of host NOD2 receptor (specifically, by priming an anti‐tumour myeloid cell response), highlighting their critical role [114]. As a second example, recent work by two groups shed light on the importance of STING (stimulator of interferon genes)‐receptor activation. Si *et al* found oral administration of *Lactobacillus rhamnosus* GG to improve anti‐PD1 efficacy *in vivo*, mediated by STING‐dependent induction of type I interferons [126]. Similarly, Lam *et al* showed that microbial cyclic dinucleotides activated STING receptors on intratumoural monocytes, generating type I interferons to shift to an anti‐tumour immune microenvironment [115]. Lastly, Toll‐like receptors (TLRs) are another important microbial pattern‐recognition receptor, with exogenous TLR agonist administration augmenting anti‐PD1 efficacy in head and neck cancers [127].

Finally, microbe–tumour molecular mimicry has been proposed as a bacterial strain‐specific mechanism for conferring ICI sensitivity. Examples abound of bacterial infections initiating and exacerbating autoimmune disease, including *Streptococcus pyogenes* triggering rheumatic heart disease or glomerulonephritis, and *Campylobacter jejuni* triggering ankylosing spondylitis or Guillain–Barré syndrome [128]. In each of these cases, structural homology between bacterial and self‐antigen epitopes leads to cross‐reactivity of T cells. It is plausible that homology between gut microbiota and tumour‐specific antigens may also occur, with immune checkpoints preventing the ensuing anti‐tumour immunity (thus released by ICIs) [129]. A retrospective analysis of long‐term pancreatic cancer survivors identified T cell‐inflamed tumours, with TILs cross‐reactive to tumour neoantigens and homologous infectious disease antigens [130]. Subsequently, Bessel *et al* identified close homology between a murine melanoma (B16.SIY) and *Bifidobacterium breve* peptide epitope (SVY). They demonstrated enhanced anti‐B16.SIY immunity in mice inoculated with *B. breve*, with causality established through recapitulation of this effect by transfer of gut microbiota and SVY‐specific T cells [131]. Finally, recent work found the TMP epitope of *Enterococcus hirae* 13144 to be immunogenic in murine cyclophosphamide recipients and strongly homologous with the *Psmb4* mouse tumour antigen. Using highly sensitive culturomics, TMP1 was found to be enriched in stool samples from RCC and melanoma anti‐PD1 recipients experiencing longer OS, providing (to our knowledge) the first clinical evidence supporting this phenomenon [113].

Together, these data demonstrate the complex mechanisms by which microbiota may influence ICI efficacy. Though early in development, there are now emerging examples of this science translating into clinically useful predictors of ICI efficacy. For example, *A. muciniphila* had previously been shown to induce Th1 CD4^+^ T cell differentiation and enhance ICI responses in murine models [92]. Based on this work, Derosa *et al* recently reported that detectable baseline stool *Akkermansia* sp. was (modestly) associated with anti‐PD1 ORR in a cohort of 338 advanced NSCLC patients (NCT04567446) – the largest such study published so far [109].

## Concluding remarks

In this review, we have attempted to highlight the key evidence supporting the significant contribution that various host factors have in the anti‐cancer efficacy of ICIs. We have covered host factors derived from the peripheral immune compartment, germline genetics, host phenotype, and the exposome (including our gut microbiome), and have discussed the strength of their implications and potential mechanisms (Table 2). It is important to emphasise that these factors are biomarker candidates only, and all require robust validation through appropriately designed, prospective clinical trials, prior to clinical implementation [132].

**Table 2:** Pre‐treatment host‐based biomarker candidates.

Category	Pre‐treatment factor	Potential assay	Possible immune mechanism	Association with ICI efficacy
Circulating immune compartment	Neutrophil–lymphocyte ratio	Blood WBC differential	Tumour‐associated neutrophils → ↓ tumour CD8^+^ T cell infiltrate	Negative [17]
	Eosinophils	Blood WBC differential	↑ Tumour CD8^+^ T cell infiltrate	Positive [21, 22, 23, 25]
	Regulatory T cells	PBMC flow cytometry	Key target of anti‐CTLA4 inhibition	Positive [22]
	Classical monocytes	PBMC flow cytometry	?	Positive [27]
	TCR repertoire diversity of PD1^+^CD8^+^ T cells	PBMC flow cytometry → sorting → targeted DNA/RNA sequencing	↑ Opportunity for tumour neoantigen recognition by cells targeted by anti‐PD1	Positive [35]
	IL‐8	Blood immunoassay	↑ Tumour neutrophil and MDSC infiltrate	Negative [39]
Germline genetics	HLA‐A*03 genotype	Blood DNA sequencing	?	Negative [44]
	HLA‐I diversity	Blood DNA sequencing	Presentation of a ↑ repertoire of neoantigens	Positive [47, 50]
	HLA‐I evolutionary divergence	Blood DNA sequencing	Presentation of a ↑ repertoire of neoantigens	Positive [48]
	HLA‐I promiscuity	Blood DNA sequencing	↓ Tumour:self discrimination → ↑ peripheral immune tolerance	Negative [53]
Body phenotype	Obesity	Body mass index	↑ Tumour % PD1^+^CD8^+^ T cell infiltrate	Positive [67]
	Cholesterol level	Blood lipid panel	↑ Tumour % PD1^+^CD8^+^ T cell infiltrate	Positive [70]
Exposome and the gut microbiome	Corticosteroids	Medical history	CD4^+^ and CD8^+^ T cell apoptosis	Negative [76]
	Antibiotics/PPIs	Medical history	↓ Favourable gut microbiota (see below)	Negative [82, 83]
	Antihistamines	Medical history	Block HRH1 → ↓ M2 macrophage differentiation	Positive [80]
	Dietary fibre intake	Medical history	↑ Favourable gut microbiota (see below)	Positive [84]
	Specific gut microbial species/subspecies	Stool metagenomic sequencing	Absorbed bacterial metabolites → immunomodulatory properties Activate innate immunity receptors on intratumoural monocytes → innate immune priming → ↑ M1 macrophage differentiation ‘Molecular mimicry’ → cross‐reactive T cells	Positive or Negative (see Table 1)

HLA, human leukocyte antigen; HRH1, histamine receptor H1; ICI, immune checkpoint inhibitor [i.e. anti‐PD(L)1 and/or anti‐CTLA4]; MDSC, myeloid‐derived suppressor cell; PBMC, peripheral blood mononuclear cell; PPI, proton pump inhibitor; TCR, T‐cell receptor; WBC, white blood cell.

Several of these candidate host factors are inherently modifiable; so, beyond their predictive biomarker potential, they may even provide avenues for improved ICI efficacy. Trials to assess this are ongoing in some cases: for example, combining ICIs with potentially beneficial concomitant medications (e.g. propranolol [133]) or strategies to modify the gut microbiome with faecal microbial transplantation, specific bacterial consortia, or single strains (recently reviewed [134]).

It is also notable that although we have discussed factors independently, we hypothesise that a multimodal approach, considering these host factors as well as tumour and tumour‐microenvironment features concurrently, might most optimally predict which patients will benefit from ICIs. Developing such an integrative, multivariate model would require sufficiently powered, richly annotated clinical datasets, while using best practices to generate high‐quality, matched tumour, host, and gut microbiome feature sets. To this end, the ongoing MITRE study (NCT04107168) seeks to enrol up to 1800 NSCLC, RCC or melanoma patients undergoing ICI therapy [135]. We hypothesise that a prospective study of this scale, integrating rich clinical and multi‐omic datasets, is necessary to confirm the relevance of host, microbial, and tumour features (either independently or in concert) to ICI efficacy, and ultimately to help progress promising candidates towards clinical use as predictive biomarkers.

## Author contributions statement

AG and DJA conceptualised the paper. AG and AMR prepared the original draft. AMR, OK, AB, TDL, SJW and DJA provided content, detailed reviews, and edits within their areas of expertise. AG and AMR created the figures. DJA and SJW provided overall supervision. All the authors provided final approval of the submitted version.
